# Patterns of self-harm presentations at a Tertiary Urban Hospital in Kenya: A retrospective 5-year study (2018–2022)

**DOI:** 10.1371/journal.pone.0317981

**Published:** 2025-01-27

**Authors:** Willie Njoroge, Catherine Gitau, Everline Onchari, Linnet Ongeri, Linda Khakali, Murad Khan, Zul Merali, Lukoye Atwoli, Jasmit Shah

**Affiliations:** 1 Brain and Mind Institute, Aga Khan University, Nairobi, Kenya; 2 Department of Medicine, Aga Khan University, Nairobi, Kenya; 3 Kenya Medical Research Institute, Centre for Clinical Research, Nairobi, Kenya; NYU Grossman School of Medicine: New York University School of Medicine, UNITED STATES OF AMERICA

## Abstract

**Introduction:**

Self-harm represents a complex and multifaceted public health issue of global significance, exerting profound effects on individuals and communities alike. It involves intentional self-poisoning or self-injury with or without the motivation to die. Although self-harm is highly prevalent, limited research has focused on the patterns and trends of self-harm among hospital populations in low- and middle-income countries, particularly within Africa. This study aims to explore the socio-demographic and clinical profile of patients presenting with self-harm and determine the common self-harm patterns at a tertiary facility in Kenya.

**Methodology:**

We carried out a descriptive retrospective study and included patients from inpatient units and outpatient settings within the Secion of Psychiatry at the Aga Khan University Hospital, Nairobi from January 1^st^ 2018 to December 31^st^ 2022. A data abstraction tool was used to collect data from eligible files sourced from the medical records department for all patients who met the study criteria. Summary statistics were reported as frequencies and percentages for categorical data and as means and standard deviations for continuous data.

**Results:**

A total of 507 files were reviewed in the given timeframe and 497 patients were included in the analysis. Of these patients, 28.1% (n = 144) presented with self-harm. The mean age of the self-harm patients was 26.5 years (SD = 10.5) and a majority (74.3%) were female. The first point-of-contact was at the emergency department in 72.9% of the cases. A majority of them, i.e. 89.6%, reported a past psychiatric diagnosis. Based on the psychiatric diagnosis evaluation of the patients- depression was the most common diagnosis at 88.2%, followed by anxiety disorder at 27.8% and bipolar mood disorder at 17.4%. The majority of reported self-harm cases involved overdose incidents (68.8%), with self-injury accounting for 56.3% of cases. Analgesics were the most frequently reported type of overdose, followed by tricyclic antidepressants. In context of self-injury, cutting emerged as the predominant form of self-harm. Family conflict was reported to be the most common reason for self-harm at 39.6%.

**Conclusion:**

This study shows a high rate of self-harm among patients with mental illness in this facility, necessitating the development of self-harm prevention and management protocols. A national registry of self-harm behavior would also help further elucidate the occurrence and mechanisms of self-harm in the population, improving the possibility for early interventions and prevention.

## Introduction

The World Health Organization (WHO) defines suicide as the intentional act of killing oneself, while self-harm is described as a non-fatal deliberate initiation of non-habitual behavior [1]. Not all instances of self-harm are linked to suicidal intent; they may serve as a means of communication, a call for help, or a coping mechanism in challenging situations or emotional distress [2]. The WHO recognizes suicide as a global public health concern, with over 700,000 death reported annually, and estimates suggest 20 to 50 suicide attempts for every suicide death. Suicide carries a particularly high burden among individuals aged 15–29, a demographic that is significant in sub-Saharan Africa. Within this age group, suicide is the fourth leading cause of death, followed by road injuries, tuberculosis, and interpersonal violence [3]. Notably, 78% of suicides worldwide occur in low-and-middle income countries (LMICs), with sub-Saharan Africa experiencing the highest burden at 11 per 100,000 population [4]. Following an act of self-harm, the risk of suicide escalates significantly, reaching 50 to 100 times the rate observed in the general population [5]. In Kenya, a LMIC, suicide is a significant concern and the WHO estimates there were 3,214 suicides and a rate of 11.0 per 100,000 deaths in 2019 [5]. With an estimated ratio of 10–20 acts of self-harm for every suicide, it is likely there are between 32140 to 64280 cases of self-harm in Kenya every year. Yet the reliability of this estimate is uncertain due to the lack of systematic data collection [3].

The impact of self-harm is multifaceted and far reaching affecting the individual, families and communities. For affected individuals and their families, the emotional and social repercussions of stigma and isolation significantly diminish social support, thereby impeding the victim’s recovery process. Additionally, the treatment cost incurred can be substantial and from a public health perspective and this can present a burden to the health care system of a nation due to the emergency, inpatient and mental health services required. The economic impact of self-harm may be as a result of the loss of productivity and income for individuals and their families.

Psychological factors are among the highest contributors to self-harm. These factors include mental health disorders, trauma and abuse. Other factors may be more pronounced in specific demographics, for example, social factors like bullying and peer pressure tend to affect the youth more, while family dynamics like dysfunctional family relationships can affect any member of the family. Socioeconomic stressors are important environmental factors, especially highlighted in LMIC populations.

Kenya is committed to ensuring that suicide prevention remains a key focus area in its public health agenda. To this end, the country has developed a National Suicide Prevention Strategy with the ambitious goal of achieving a 10% reduction in suicide mortality by 2026 [6].This strategy is built on five key pillars:1) establishing intersectoral committees on suicide prevention at both national and county levels to coordinate efforts, 2) strengthening policies to facilitate effective implementation of suicide prevention programs, including advocating for the decriminalization of suicide, 3) enhancing access to comprehensive, integrated, and quality suicide intervention services across all levels of care, 4) increasing awareness and reducing stigma surrounding suicide through education and advocacy, and 5) strengthening surveillance and research to better understand and address suicide and its associated factors [6].

Despite these efforts, suicide remains highly stigmatized in Kenya, similar to other sub–Saharan African (SSA) countries, and is often considered a taboo subject. Many Kenyans attribute suicide to supernatural or spiritual causes [7]. Gender disparities are notable: while more females attempt suicide, males are more likely to die by suicide, often due to the use of more lethal methods. The motivations for self-harm also exhibit gendered patterns. Men are more likely to self-harm due to financial stress and societal pressure to fulfill their roles as breadwinners, while women are often triggered by intimate partner violence and relationship discord [8].

Kenya is yet to establish a national suicide and self-harm surveillance system and therefore its estimates of this burden may not be reliable. This lack of data impedes the prioritization and planning of suicide prevention strategies. To inform the development of a national self-harm and suicide registry, a pilot self-harm registry was established at Aga Khan University Hospital in Nairobi in 2023. This initiative also leveraged the opportunity to analyze retrospective self-harm data from the facility before the registry’s establishment. This paper focuses on a five-year retrospective analysis of this data.

This study aims to explore the prevalence of self-harm, describe the socio-demographic and clinical profile of patients presenting with self-harm and determine the common self-harm methods at a tertiary facility in Kenya. In addition, the study seeks to determine demographic associations among individuals presenting with self-harm compared to those who do not present with self-harm.

## Methods

We carried out a descriptive retrospective cohort study from January 1^st^, 2018, to December 31^st^, 2022, and records of all inpatients and outpatients who presented to the psychiatry clinic or the Accident & Emergency (A&E) ​Department at the Aga Khan University Hospital, Nairobi (AKUHN) for any mental health condition were analyzed. The hospital is a private, fee-for-service but not-for-profit institution that provides tertiary and secondary level health care services. It has a 300-bed capacity and over 50 outreach medical centers across East Africa. The data was obtained within the medical records department from 20^th^ February 2023 to 20^th^ July 2023 for this research study.

We developed a data abstraction tool incorporating relevant variables identified through a comprehensive review of the literature and routinely collected data from patients’ medical records. The data abstraction tool was validated by the research team, mainly those involved in the Psychiatry department. This variables included in the data tool captured demographic data such as age, gender, location (site where the patient presented), time of presentation, religion, residence, marital status, occupation, education level and clinical data; method of overdose, method of self-poisoning, reason, intent, past psychiatric diagnosis, medical diagnosis, previous attempts, psychiatric medicine prescribed, psychiatric plan and follow up, duration of hospital stay, any contact with healthcare prior to self-harm. The inclusion criteria encompassed all inpatients and outpatients who presented at the Psychiatry Clinic or A& E Department at the AKUH, Nairobi for any mental health condition.

The data was collected using the Research Electronic Data Capture (REDCap) platform (Vanderbilt and National Institute of Health) [9]. Self-harm was defined as intentional actions that result in physical injury to oneself, regardless of whether there is an intent to die. Self-harm is not specifically coded in the ICD system and was therefore identified through a manual review of all eligible medical records. The key terms used to identify was “self-harm”, “suicide attempts”, “poisoning”, “overdose”, and “cutting” were manually checked to identify cases of self-harm. The data collection was done by a research assistant (EO) who was trained in collecting the data from medical records. The retrieved medical records were assessed for inclusion by a psychiatrist (CG) and the research assistant (EO), using the pre-specified inclusion/exclusion criteria. Data was extracted from medical files by the research assistant into a tailored data extraction tool and entered into REDCap. All the data extraction was checked by two reviewers (WN and JS) to ensure that all the fields had been filled and any discrepancies were resolved by consensus discussion. Approval for this study was obtained from the Institutional Scientific and Ethics Review Committee (ISERC) at the Aga Khan University, Nairobi (Ref: 2022/ISERC-135(v1)) and National Commission for Science Technology and Innovation (NACOSTI) (NACOSTI/P/23/23187).

Continuous data were summarized using means and standard deviations (SD), whereas categorical data were analyzed as frequencies and percentages. Group comparisons were analyzed using univariate analysis using Fishers Exact test for categorical data and Kruskal Wallis data for continuous data. A p-value of less than 0.05 was considered statistically significant. All data analysis was performed using SPSS statistical software V. 20.0 (IBM, Armonk, NY, USA).

## Results

A total of 507 patients files were reviewed, 497 were included in the analysis, and 10 files were excluded due to missing data. Out of the 497 patient files, 29.0% (n = 144) documented cases of self-harm whereas the remaining 71.0% (n = 353) were not cases of self-harm. The overall demographic characteristics of the 497 patients are presented in Table 1 in comparison with those presenting with self-harm.

**Table 1:** Demographic characteristics of all patients and in comparison, with self-harm and non-self-harm patients.

		Total		Yes Self-Harm		No Self-Harm		P Value
		(N = 497)		(n = 144)		(n = 353)		
Age (years)		34.4 [13.7]		26.5 [10.5]		37.6 [13.5]		<0.001
Year of Presentation	2018	49	9.9%	8	5.6%	41	11.6%	0.054
	2019	93	18.7%	27	18.8%	66	18.7%	
	2020	85	17.1%	18	12.5%	67	19.0%	
	2021	113	22.7%	39	27.1%	74	21.0%	
	2022	157	31.6%	52	36.1%	105	29.7%	
Gender	Male	241	48.5%	37	25.7%	204	57.8%	<0.001
	Female	256	51.5%	107	74.3%	149	42.2%	
Location (Site)	ER	214	43.1%	105	72.9%	109	30.9%	<0.001
	OPD	283	56.9%	39	27.1%	244	69.1%	
Religion Type (n = 488)	Christian	403	82.6%	122	90.4%	281	79.6%	0.015
	Muslim	42	8.6%	8	5.9%	34	9.6%	
	Others	43	8.8%	5	3.7%	38	10.8%	
Occupation	Student	125	25.2%	73	50.7%	52	14.7%	<0.001
	Employed/Self-employed	297	59.8%	63	43.8%	234	66.3%	
	Other	75	15.1%	8	5.6%	67	19.0%	
Education level (n = 493)	Primary / Secondary	94	19.1%	40	27.8%	54	15.5%	<0.001
	College	269	54.6%	60	41.7%	209	59.9%	
	Graduate / Postgraduate	130	26.4%	44	30.6%	86	24.6%	
Marital Status	Single	245	49.3%	108	75.0%	137	38.8%	<0.001
	Married	207	41.6%	30	20.8%	177	50.1%	
	Other	45	9.1%	6	4.2%	39	11.0%	
Past Psychiatric Diagnosis	Yes	247	49.7%	129	89.6%	118	33.4%	<0.001
	No	250	50.3%	15	10.4%	235	66.6%	

Younger age and female gender were associated with greater self-harm and this was statistically significant. The mean age of patients who presented with self-harm was 26.5 years (SD = 10.5) as compared to 37.6 years (SD = 13.5) of those not presenting with self-harm. Marital status was associated with self-harm, where 75.0% of those presenting with self-harm were single as compared to only 38.8% of ones who reported their marital status as single and not presenting with self-harm (p<0.001). Furthermore, past psychiatric diagnosis was associated with a greater risk of self-harm, where 89.6% of those that presented with self-harm had a past diagnosis, whereas only 33.4% of those presenting with no self-harm had a past diagnosis. The trends of presentation increased over time for both the self-harm and no self-harm group. In the self-harm group, the proportions rose from 5.6% in 2018 to 36.1% in 2022, while in the no self-harm group, it increased from 11.6% to 29.7% over the same period. The overall comparison between self-harm versus no self-harm is presented in Table 1.

**Fig 1:**
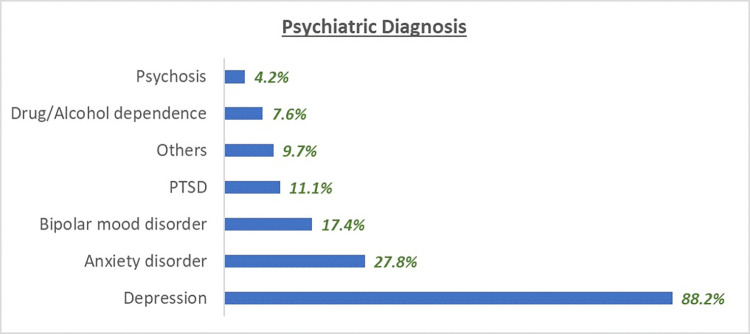
Psychiatric diagnosis on evaluation at presentation of self-harm patients (n = 144).

For those that reported with self-harm, only 19.4% reported not having a previous attempt, whereas 29.2% reported having one previous attempt and 51.4% reported having more than one previous attempt. The psychiatric diagnosis for those who presented with self-harm is presented in Fig 1. A patient could have been diagnosed with more than one condition as reported in the medical records.

### Reasons and patterns of Self-harm

More than half reported having an overdose at 68.8% and the most common type of overdose was analgesics (54.5%), followed by tricyclic antidepressants (25.3%). Self-injury was reported at 56.3%, with cutting being the most prevalent type of self-injury (75.3%) and other forms, such as choking, stabbing, hitting, drowning etc. (16.0%). Only 21.5% reported self-poisoning with alcohol poisoning being the most prevalent type (32.3%) followed by organophosphate / pesticide poisoning (29.0%). The leading cause of self-harm was family conflict reported by 41.0% of the cases, followed by exam or studies related stress (18%) and other relationship issues (18%). Based on intent—50.0% reported to die and 38.2% reported getting out of a situation. The detailed reasons and patterns are presented in Table 2.

**Table 2:** Reasons and patterns of self-harm patients.

Overdose	Yes	99	68.8%
	No	45	31.3%
Overdose	Benzodiazepine	20	20.2%
	Tricyclics (amitriptyline)	25	25.3%
	Antipsychotics/Barbiturates	6	6.1%
	Selective serotonin reuptake inhibitors	10	10.1%
	Analgesics	54	54.5%
	Other medicine	10	10.1%
Self-injury	Yes	83	57.6%
	No	61	42.4%
Self-injury	Cutting	61	75.3%
	Burning	4	4.9%
	Hanging	11	13.6%
	Jumping (height / traffic)	11	13.6%
	Others	13	16.0%
Self-poisoning	Yes	31	21.5%
	No	113	78.5%
Self-poisoning	Organophosphate / pesticide	9	6.3%
	Bleach / detergent	7	4.9%
	Rat poison	2	1.4%
	Alcohol poisoning	10	6.9%
	Others	4	2.8%
Reasons for self-harm (n = 139)	Marital issue	23	16.5%
	Family conflict	57	41.0%
	Financial	10	7.2%
	Work stress	15	10.8%
	Exam/studies	25	18.0%
	Medical or Psych illness	18	12.9%
	Mistake	5	3.6%
	Other relationship issue	25	18.0%
	Bereavement	9	6.5%
	Others	40	28.8%
Intent	To die	72	50.0%
	To put pressure/ force a decision	22	15.3%
	Sleep off	36	25.0%
	To get out of a situation	55	38.2%
	Impulsive act	19	13.2%
	Seek attention	10	6.9%
	Others	4	2.8%
Farewell act	Yes	7	4.9%
	No	136	94.4%
	Not known	1	0.7%
Previous Attempts	None	28	19.1%
	Once	42	29.2%
	Multiple	74	51.4%

### Spiritual history of self-harm patients

Documentation of spiritual history was available from 135 patients with documented self-harm. Of these, 90.4% reported being currently active in their faith and associated community and 85.2% reported being part of a religious or spiritual community. Furthermore, 87.4% reported support of their faith being available to the patients whereas 79.3% reported whose presence and support they value at a time like this. Detailed characteristics of spiritual history are presented in Table 3.

**Table 3:** Self-harm patients’ spiritual history.

Are you currently Active in your faith community?	Yes	122	90.4%
	No	13	9.6%
Are you part of a religious or spiritual community?	Yes	115	85.2%
	No	20	14.8%
Is support for your faith available to you?	Yes	118	87.4%
	No	17	12.6%
Is there a person or a group whose presence and support you value at a time like this?	Yes	107	79.3%
	No	28	20.7%
Is your faith (your beliefs) helping you Cope?	Yes	115	85.2%
	No	20	14.8%
Do any of your religious beliefs or spiritual practice Conflict with medical treatment?	Yes	3	2.2%
	No	132	97.8%
Are there any particular concerns you have for us as your medical team?	Yes	1	0.7%
	No	134	99.3%
Recommend Spiritual Counselling	Yes	5	3.7%
	No	130	96.3%

### Gender differences among self-harm

Based on gender differences, age was a significant factor, where females presented at a younger age as compared to males (25.4 years vs 29.7 years; p = 0.040). Depression and anxiety disorders was more prevalent in females as compared to males, but this was not statistically significant. Overdose was higher in females compared to males and this was statistically significant (73.8% vs 54.1%, p = 0.039). In contrast, self-injury and self-poisoning were reported to be higher in males as compared to females, but was not statistically significant. The differences between males and females with the variables are summarized in Table 4.

**Table 4:** Gender differences among various self-harm variables.

		Male		Female		*P* Value
		(n = 37)		(n = 107)		
Age (years)		29.7 [11.6]		25.4 [9.9]		0.040
Marital Status	Single	25	67.6%	83	77.6%	0.286
	Married	11	29.7%	19	17.8%	
	Other	1	2.7%	5	4.7%	
Psychiatric Diagnosis	Depression	30	81.1%	97	90.7%	0.142
	Anxiety disorder	6	16.2%	34	31.8%	0.089
	Bipolar mood disorder	9	24.3%	16	15.0%	0.213
Overdose	Yes	20	54.1%	79	73.8%	0.039
	No	17	45.9%	28	26.2%	
Self-injury	Yes	22	59.5%	59	55.1%	0.703
	No	15	40.5%	48	44.9%	
Self-poisoning	Yes	11	29.7%	20	18.7%	0.170
	No	26	70.3%	87	81.3%	
Past psychiatric diagnosis	Yes	33	89.2%	96	89.7%	1.000
	No	4	10.8%	11	10.3%	
Previous attempts	Yes	28	75.7%	88	82.2%	0.470
	No	9	24.3%	19	17.8%	
Reasons for self-harm	Marital issue	6	16.2%	17	15.9%	1.000
	Family conflict	16	43.2%	41	38.3%	0.697
	Financial	2	5.4%	8	7.5%	1.000
	Work stress	5	13.5%	10	9.3%	0.534
	Exam/studies	6	16.2%	19	17.8%	1.000
	Psych illness / Medical illness	8	21.6%	10	9.3%	0.080
	Mistake	2	5.4%	3	2.8%	0.603
	Other relationship issue	3	8.1%	22	20.6%	0.129
	Bereavement	4	10.8%	5	4.7%	0.235
	Others	10	27.0%	30	28.0%	1.000

## Discussion

Our retrospective facility-based study revealed several key findings regarding self-harm patients. The majority were female, younger in age, single, and had attained a higher level of post-secondary education. Of particular concern were the high rates of self-harm attempts among individuals and the elevated levels of suicidal intent observed. The most commonly reported self-harm method reported was overdose, notably involving analgesics and tryclics antidepressants.

Additionally, relationship issues were identified as a common trigger for self-harm in other studies [10]. Our study observed an increasing trend in self-harm cases among young people, with the age of onset decreasing to an average of 10.8 years. Notably, the study documented two cases of self-harm in children aged 10 and 11 years. Social and economic issues such as marital problems, work-related stress, financial difficulties, romantic relationship challenges, and family conflicts were identified as the primary triggers for patients engaging in self-harm. The highest reported intention behind self-harm was a desire to die, accounting for 50% of cases. However, only 4.9% of self-harming patients performed farewell acts, such as writing a note, suggesting that many instances were impulsive acts, cries for help, or coping mechanisms rather than planned suicide attempts. Of the self-harm cases reported, 74.3% were females. Liddon et al presented the higher female representation aligns with the tendency for women to seek medical care more frequently and spend more time in consultations [11]. In general, females tend to excel in recognizing emotions and articulating their feelings more readily. Additionally, they exhibit higher levels of participation in treatment and are more proactive in reporting mental health symptoms [12,13].

Majority of patients (80%) had a history of previous self-harm attempts, indicating a heightened likelihood of recurrence for this group. All patients (100%) received medical attention and 99.3% prescribed psychiatric medication. The fact that only one patient left against medical advice suggests the quality of care provided by the hospital.

Our findings indicate that while religious affiliation may not act as a protective factor against self-harm, it does offer protection against self-harm attempts. The effectiveness of religious affiliation in preventing suicide attempts could be influenced by culture-specific implications, particularly for minority religious groups who might experience social isolation. Our findings on the role of religion and spiritual history provide a basis for creating more targeted educational interventions. Therefore, conducting a spiritual history is essential not only to uncover spiritual resources that can support psychological well-being, but also to identify capabilities that may directly influence suicide prevention. A spiritual history can be seamlessly integrated into the social history during hospital admission, new patient evaluations, or outpatient visits [14]. Moreover, addressing spiritual matters has been shown to improve the doctor-patient relationship and foster trust [15]. When spiritual needs are recognized, interventions based on spirituality can be introduced. These interventions are designed to help clinicians guide religious patients in accessing supportive religious resources. Additionally, they promote increased awareness among religious communities and leaders about beneficial practices, fostering the provision of more support within religious settings.

Some targeted strategies can be employed to address aspects of relationship discord and academic stress. Family conflict resolution initiatives, such as community-based counseling led by non-specialists, including clergy [16]. School-based mental health programs can address academic stress by equipping both teachers and pupils with tools to promote mental wellness and practice wellbeing strategies [17,18]. Moreover, mental health stigma, particularly around suicide, has a significant impact on health-seeking behavior [19]. This stigma is especially pronounced among men, who are culturally expected to remain stoic and "masculine." These societal norms discourage men from expressing vulnerability or seeking help, whereas emotional expression is more culturally acceptable for women [20,21]. Addressing these cultural barriers is essential to fostering healthier attitudes toward mental health and encouraging timely intervention.

### Strengths and limitations of the study

The use of routine data is both a strength and a limitation of this study. Since the study depended on medical records that were originally not designed to collect data for research, some information was bound to be missing. Selection and recall biases could also impact the results and reasons for differences in treatment between patients and lost follow ups cannot be ascertained and could lead to biases. AKUHN is a private hospital and patients presenting here may not be truly representative of the general population of Nairobi or Kenya. Despite the limitations, using a linkage of routinely collected data provides a fast, less costly, and ethically viable research option compared with conducting a prospective prevalence study of self-harm patients in contact with health care workers. Additionally, both mental illness and self-harm are potentially stigmatizing areas of health which are likely to influence participation in any primary data collection research. The use of routinely collected data improves representativeness of the sample by reducing bias due to sampling selection and bias due to missing data.

## Conclusion

Our retrospective study, conducted at a tertiary care facility, yielded several significant findings pertaining to patients presenting with self-harm behaviors. The majority were younger females, single college students. Notably, there were high rates of repeated self-harm attempts and elevated levels of suicidal intent. Overdose was the most frequently reported method of self-harm, specifically involving analgesics and tricyclic antidepressants. Based on the high self-harm burden found in this study population, we recommend routine screening and assessment for general hospital patients and training for all frontline health care workers on self-harm management protocols. Finally, we advocate for the cautious and regulated use of tricyclics antidepressants in patients at risk of self-harm. Future studies can examine health care utilization including follow up care patterns as well as treatment approaches for self-harm management.

## Supporting information

S1 Table This is the data for the Self harm patients. (XLSX)

S2 Table This is the data for the No Self harm patients. (XLSX)
